# The Incidence and Characteristics of Pelvic-Origin Varicosities in Patients with Complex Varices Evaluated by Ultrasonography

**DOI:** 10.3390/tomography10070088

**Published:** 2024-07-19

**Authors:** Kwon Cheol Yoo, Hyung Sub Park, Chang Sik Shin, Taeseung Lee

**Affiliations:** 1 College of Medicine, Chungbuk National University, Cheongju 28644, Republic of Korea; ykc1019@naver.com; 2 Department of Surgery, Chungbuk National University Hospital, Cheongju 28644, Republic of Korea; 3 Department of Surgery, Seoul National University Bundang Hospital, Seongnam 13620, Republic of Korea; 4 Department of Surgery, College of Medicine, Seoul National University, Seoul 03080, Republic of Korea; 5 Department of Surgery, Uijeongbu Eulji Medical Center, Uijeongbu 11759, Republic of Korea

**Keywords:** varicose veins, gonadal vein reflux, ultrasonography, pelvic region

## Abstract

Objective: The purpose of this study was to evaluate the incidence of gonadal vein refluxes associated with lower-extremity varicose veins with Doppler ultrasonography (DUS). Method: A total of 6279 patients with venous disease-related symptoms of the lower extremity were evaluated with DUS in the vascular lab. Gonadal vein reflux using abdominal ultrasound was further evaluated in patients with unusual varices, defined as varices in the inguinal, inner or upper thigh and the vulvar area without refluxes in the saphenofemoral junction (SPJ). Those patients who showed gonadal vein reflux were diagnosed as having pelvic-origin varicosity. Results: Unusual varices were found in a total of 237 patients (3.8%), and of these patients, pelvic-origin varicosity was discovered with transabdominal ultrasound in 156 (65.8%). A total of 66.7% (n = 38/57) of unusual varix patients with pelvic pain had gonadal vein reflux. The measurement of gonadal vein diameter was larger in ultrasonography than CT scans (8.835 vs. 8.81, *p* < 0.001). Two patients with severe symptoms but no obstructive venous diseases were treated with gonadal vein embolization. Conclusion: The incidence of pelvic-origin varicosities was 2.5% (n = 156/6279). However, more than half of the patients with unusual varices had gonadal vein reflux and 24.4% of these patients also presented with pelvic pain. The evaluation of pelvic-origin varicosities should be performed in patients who present with unusual forms of varices of the lower extremity.

## 1. Introduction

Varicose vein is a common medical disease with a reported prevalence of approximately 23% in adults in the United States. The reported prevalence of chronic venous diseases that result in skin color changes or venous ulcers is 6% [1]. In addition to cosmetic problems, varicose veins can cause lower leg discomfort and pain that result in limitations in movements and poor quality of life [1,2,3]. In severe cases with chronic venous disease, ulcers can result in infections that may even lead to amputation [4].

Several primary and recurring varicose veins of the lower limb have been reported to be caused by pelvic venous insufficiency and should not be overlooked [5]. Especially in women, pelvic venous insufficiency has been reported in 4 to 15% of varicose vein patients [6,7]. However, there is a lack of reporting in Eastern countries. In cases of pelvic venous insufficiency, one cause of chronic pelvic pain is the malfunction of the gonadal or hypogastric vein. This is sometimes referred to as pelvic congestion syndrome [8]. 

Chronic pelvic pain represents a prevalent reason for gynecological outpatient visits [9], with a global prevalence ranging from 4% to 43%. Amongst women, it affects a substantial number, accounting for between 16% and 31% [10]. The clinical manifestations of chronic pelvic pain vary significantly, from asymptomatic to severe conditions [11].

There are three major differential diagnosis that can be made for chronic pelvic pain. It can be caused by reflux due to pelvic vein incompetence, the obstruction of venous outflow in the iliac or renal vein and finally gynecological diseases such as endometriosis. The purpose of this article is to investigate the incidence and characteristics of gonadal vein reflux in patients with low-extremity varicose veins by evaluation through Doppler ultrasonography (DUS).

## 2. Methods

### 2.1. Study Design

From January 2010 to February 2024, we retrospectively analyzed patients who underwent duplex ultrasonography under the suspicion of varicose veins of the lower extremities. Ultrasonographic evaluation of both lower limbs was performed routinely. Patients with unusual varices were suspected of gonadal vein refluxes and further evaluation via abdominal ultrasound was undertaken. Unusual varices were defined as varices in the inguinal area, inner or upper thigh and vulvar area without the presence of reflux in the saphenofemoral junction (SFJ). These unusual varix patients who showed gonadal vein reflux were diagnosed as having pelvic-origin varicosity.

### 2.2. Low-Extremity DUS

A pulsed-wave 7.5 MHz Doppler with linear array transducers (Philips, Amsterdam, The Netherlands) was used for evaluating the saphenofemoral junction (SFJ), saphenopopliteal junction (SPJ), great saphenous vein (GSV), small saphenous vein (SSV), femoral vein (FV), popliteal vein (PV) and tibial vein (TV) in both lower extremities. The subjects were positioned in the 30~45-degree reverse Trendelenburg position with the leg rotated outward and weight taken on the opposite leg. For measuring refluxes in the distal limb, provocation was induced by manual compression of the calf muscle. When refluxes were checked in proximal SFJ and FV, provocation was induced by a 2 s duration Valsalva maneuver [12,13,14,15,16,17].

The examination started below the inguinal ligament, and included superficial and deep veins in 3~5 cm interval lengths. Five standardized sites were routinely checked in the saphenous vein: (1) the inguinal ligament, 2 cm distal to the SFJ; (2) above the femoral epicondyle; (3) the popliteal fossa, at least 2 cm distal to the saphenopopliteal junction (SPJ); (4) below the tibial epicondyle of the tibia; and (5) the lower one-third of the lower leg.

### 2.3. Gonadal Vein DUS

When unusual varices were confirmed by physical examination by the physician, abdominal duplex sonography was performed to evaluate the presence of reflux in both gonadal veins.

A 2–5 MHz pulsed-wave Doppler with curvilinear array transducers (Philips, Amsterdam, The Netherlands) was used for evaluating the gonadal veins in the abdomen. The subjects were positioned in a 30~45-degree reverse Trendelenburg position.

The sonographer found the gonadal vein located anterior to the psoas muscle and lateral to the aorta and then traced it to the IVC or left renal vein confluence.

**Figure 1:**
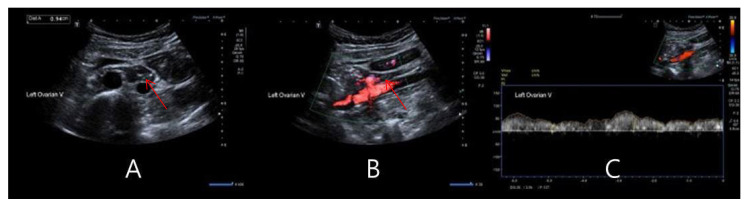
Ovarian vein duplex ultrasonography and measurement of reflux flow and time. (**A**) Transverse plane of left ovarian vein (red arrow), (**B**) longitudinal plane with color Doppler of left ovarian vein (red arrow), and (**C**) reflux time measurement of left ovarian vein.

The size of the gonadal vein was checked in a transverse view, and the transducer was rotated to hold a longitudinal view of the gonadal vein. The subjects were told to hold their breath and perform the Valsalva maneuver when checking the reflux flow and velocity of the gonadal vein (Figure 1).

### 2.4. Cut-off Values for Significant Reflux

Table 1 shows the cut-off times for significant reflux. The significant reflux time was defined as 1000 ms in the femoral vein, popliteal vein and gonadal vein. The significant reflux time in the great saphenous vein (GSV), small saphenous vein (SSV), tibial vein, deep femoral vein and perforating vein was 500 ms [18,19].

Table 2 shows the distribution of the clinical manifestations of patients presenting with unusual varices. A total of 57 patients presented with accompanying pelvic pain and of these patients, 66.7% (n = 38) had gonadal vein reflux.

Junctional reflux was defined as patients who showed SFJ, SPJ or perforating vein refluxes in DUS. Non-junctional reflux was defined as patients who showed refluxes in the superficial vein without reflux in the SFJ, SPJ and perforating veins. 

### 2.5. Computer Tomographic (CT) Imaging of the Gonadal Vein

If a patient presented with severe pelvic pain with continuous reflux in a gonadal vein over the diameter of 8 mm, and/or presented combined extravascular symptoms such as hematuria, CT venography was undertaken to evaluate the obstructive mechanisms of the vein. Differential diagnoses for obstructive mechanisms such as Nutcracker Syndrome or May–Thurner syndrome were made through CT imaging [20,21].

## 3. Results

**Figure 2:**
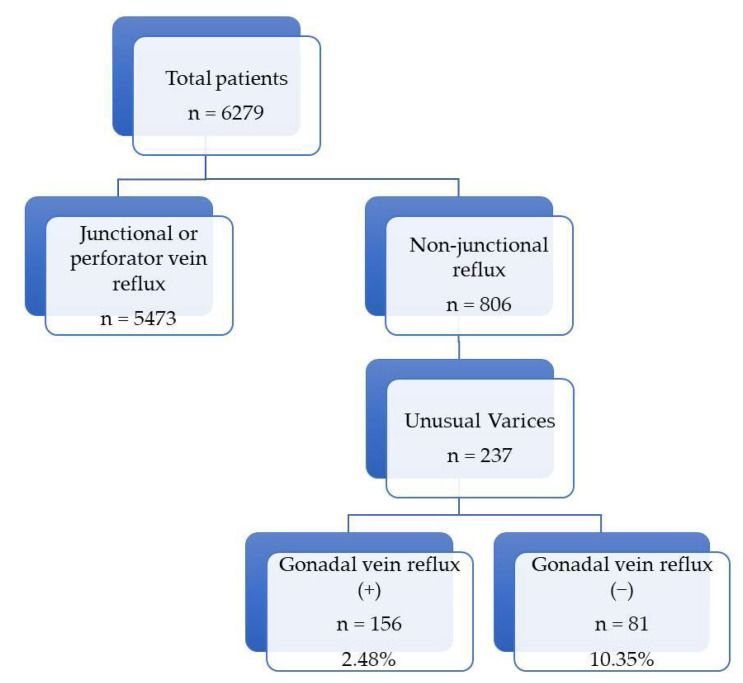
Flow chart of study population by reflux pattern.

A total of 6279 patients, who presented with lower-extremity discomfort in the out-patient clinic, were diagnosed as having varicose vein by DUS. The median age was 53 years (range 9.2–97.1 years) and 67.8% (n = 4254) of patients were female. The study population flow chart and the reflux pattern are shown in Figure 2. Junctional refluxes were found in 87.2% (n = 5473) and non-junctional refluxes were found in 12.8% (n = 806). Among non-junctional reflux patients, unusual varices were found in 237 patients. Of these patients, 65.8% (n = 156) showed gonadal reflux. Resultingly, the incidence of pelvic origin varicosity was 2.5% (n = 156/6279).

The patients with unusual varices were distinguished by the presence or absence of gonadal venous reflux, as illustrated in Table 1. However, no statistical significance was found for any of the variables. The variables include the following surgical types: high ligation and stripping of saphenous vein: 13 cases; radiofrequency ablation: 3 cases; sclerotherapy: 10 cases; and percutaneous ovarian embolization: 2 cases. 

**Figure 3:**
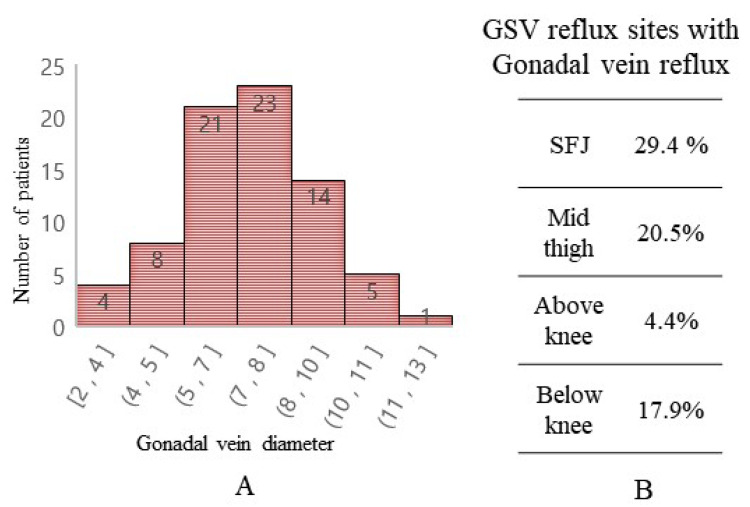
Characteristics of gonadal vein reflux. (**A**) Distribution of patients by gonadal vein diameter. (**B**) Sites of reflux in relation to GSV.

The mean diameter of the gonadal veins of patients with gonadal reflux was 7.0 ± 1.8 mm, and most of them were distributed within the range of 5 to 10 mm (Figure 3A). In addition to refluxing directly to the SFJ, there can be a back-flow to the mid-thigh, above the knee or below the knee on GSV by the collateral veins (Figure 3B).

**Figure 4:**
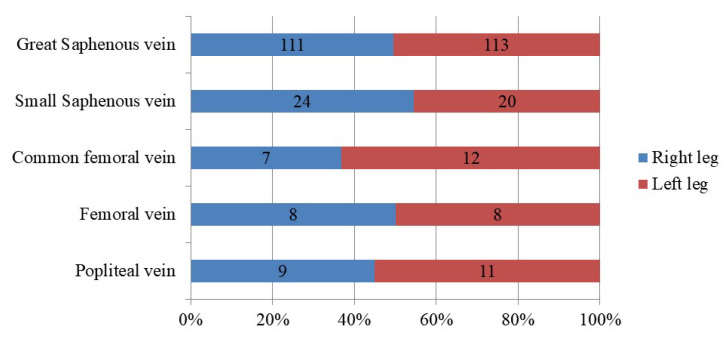
The distribution of associated lower limb venous insufficiencies with pelvic-origin varicose veins.

Except for one patient with bilateral gonadal vein reflux, all patients had reflux in the left gonadal vein only. The distributions of associated lower-limb venous insufficiencies with pelvic-origin varicose veins are shown in Figure 4. 

Additional CT imaging was performed in 20 cases. In three of these cases, Nutcracker Syndrome was suspected in the CT image. CT and sonographic diagnostic modalities were compared. The diameter of the gonadal veins was significantly larger in ultrasonography than in CT (7.835 ± 2.136 vs. 7.81 ± 1.808; *p* < 0.001 analyzed by independent-samples *t*-tests) 

One patient with bilateral gonadal venous reflux underwent ligation and stripping on the right leg. Of the 18 patients with left ovarian venous reflux, 13 underwent surgery on the right leg, 4 on both legs and 1 on the left leg (Table 3). Two of the nineteen patients who underwent surgery had additional gonadal vein embolization performed. The ultrasonographic findings of these patients showed a gonadal vein diameter of 8.8 mm and no other obstructive lesions such as Nutcracker Syndrome upon CT scanning. The remaining patients, 87.8% (n = 137), were treated with conservative treatment with medication and pantyhose-type compression stockings.

## 4. Discussion

The incidence of gonadal vein reflux in varicose vein patients was 2.5%. The female incidence was about 4% in our hospital, which contrasted with the reported 15% prevalence in Western countries [5,6]. This difference may be caused by differences in body mass index or different pelvic anatomy between races.

Pelvic vein reflux is frequently associated with combined lower-extremity venous insufficiency. The left gonadal vein was the dominant incompetent vessel, but refluxes of both sides of the limb were observed. The pelvic venous system consists of two networks, visceral and parietal networks, both of which are highly variable and are often interconnected [8,22]. If incompetence of the vein occurs in the pelvic cavity, it may lead to problems throughout the entire connected network system rather than a single vessel. In the end, laterality of a single gonadal vein reflux may not result in an ipsilateral limb insufficiency, but may affect both sides of the limb’s venous system.

Regardless of the presence or absence of reflux in the gonadal vein, patients with unusual varices showed similar clinical and morphologic patterns. Gonadal vein insufficiency itself may not affect the severity of lower-extremity venous insufficiency. However, reflux of the gonadal vein can affect the common femoral vein or saphenofemoral junction. Not all the refluxes may be directly connected to the saphenofemoral junction, but the refluxes may drain through the Giacomini vein or other veins in the posterior or inner thigh. They are connected and drained into the great saphenous vein in the mid-thigh or, below this level, even into the small saphenous vein. If reflux in the saphenofemoral junction is absent, but there is a visible reflux in the distal area, careful tracing of the reflux should be performed to find the origin of the unusual reflux. Therefore, evaluations of pelvic-origin varicosities should be performed in patients presenting with unusual varices of the lower extremity.

There were differences in diagnosing modalities. CT scans had more benefits for evaluating obstructive mechanisms and showed associations with extravascular structures. It also had less bias in detecting diseases than ultrasonography, which depends on the skill of the practitioner. Yet, ultrasonography has several advantages. It can detect the reflux of vessels, including the velocity and direction of reflux flow. However, vein sizes are often larger in sonography than CT scans. There are two main reasons why the diameter of the gonadal vein was larger in ultrasonography. First, the position of the subject was different. In CT scans, the supine position was used, but in ultrasonography, the reverse Trendelenburg position was used. This could cause venous congestion in the pelvic cavity that leads to the dilation of the gonadal vein. Second, there was a difference in provocation method. For CT scans, the examination was carried out simply by holding the breath, but the patients undergoing ultrasonography underwent the Valsalva maneuver to induce pelvic venous congestion. This may also affect the diameter of the gonadal vein.

Most pelvic-origin varicose patients had conservative treatment with medication and compressive stockings. Most conservative treatments improved patient symptoms. However, patients who had severe heaviness and varicosities in the lower extremities underwent surgery. 

This study has limitations as a retrospective analytical study. Firstly, from a hemodynamic perspective, the analysis was not designed to identify a potential source of reflux from one of the leaking points within the pelvic network. The existing literature on pelvic network leak points provides a basis for developing effective treatment strategies [23]. Two cases of perineal veins draining into the superficial veins of the perineum (labial veins and perineal fascia) and two cases of perineal veins draining from the round ligament of the uterus into the vulva and perineum have been identified [24]. All varicoceles were treated with an ultrasound-guided approach based on functional anatomy. In 11 cases [25], the drainage of the bulbous vein to the clitoris was treated by focusing on the point of leakage, with favorable outcomes. The treatment points were classified as the P-point of the perineal vein and the I-point of the inguinal ligament. Additionally, the prolapse points of pelvic-derived varicose veins were subdivided to facilitate targeted treatment. Sclerotherapy with 0.5 and 2 mL of an appropriate concentration of 1% polidocanol or 0.5% to 3% sodium tetradecyl sulfate using either liquid or foam is often performed, or pelvic vein embolization is attempted in cases of refractoriness to such treatment. For other causes, such as venous compression syndrome, stenting to reduce venous plexus hypertension is also described. The implementation of individualized treatment strategies for leak points will result in a reduction in unnecessary interventions while simultaneously maintaining the functionality of normal great saphenous vein branches. In a large-scale study of 985 women [26], 76.8% of patients identified lower-limb varicose veins caused by pelvic escape points and showed symptomatic improvement by point (perineal point: 70.8%, inguinal point: 20.7%, inferior gluteal point: 3.7%, obturator point: 3.2%, superior gluteal point: 1.6%). Echo-guided direct foam sclerotherapy and venous reflux in the pelvic region showed an improvement of symptoms by treatment through second-level imaging, and it was found that if the study had been conducted with a detailed classification of leaking points, it could have been very helpful in preserving the normal function of the great saphenous vein, and it may be a limitation of this study, which was conducted retrospectively.

Secondly, the recurrence of varicose vein surgery constitutes a risk factor. However, a history of abdominal surgery is also a risk factor, and the relevant details were not collected. Pelvic venous reflux represents the primary etiology of recurrent varicose veins following open surgical procedures [27]. The investigation of pelvic varicose veins in women presenting with symptomatic varicose veins, wherein reflux is observed from the pelvis into the leg vein pattern, should be conducted with a duplex ultrasound that incorporates a comprehensive examination of the ovarian and iliac veins to facilitate the diagnosis and treatment of the underlying cause.

Finally, the gonadal vein, which is the main source of pelvic venous reflux, is strongly influenced by sex hormones and is the main cause of cyclic pelvic pain. Sex hormones stimulate and influence the dilation of the vessels depending on their concentration, which can cause pelvic venous reflux [28]. However, there was a lack of analysis of the causative factors of reflux, and it is believed that future studies could improve this research by including recurrent patients in the study.

## 5. Conclusions

Pelvic-origin varicosity is uncommon in patients suspected of low-extremity varicose veins. However, a high percentage of patients who present unusual varices have accompanying gonadal vein refluxes and many of these patients experience pelvic pain. Therefore, the evaluation of pelvic-origin varicosities should be performed in patients who present with unusual forms of varices of the lower extremities.

## Figures and Tables

**Table 1:** Demographics and characteristics of the patients with unusual varices.

	Gonadal Vein Reflux (+) (n = 156)	Gonadal Vein Reflux (−) (n = 81)
**Sex differences (M/F)**	1/155	1/80
**Age**	53.69 ± 12.53	53.03 ± 13.05
**Height**	158.02 ± 7.61	158.02 ± 6.70
**Weight**	58.52 ± 8.21	60.90 ± 8.48
**BMI**	23.56 ± 3.72	24.43 ± 3.45
**Risk Factor**		
**No**	129 (82.6%)	58 (71.6%)
**Pregnancy**	8 (5.1%)	6 (7.4%)
**Deep vein thrombosis history**	0 (0%)	3 (3.7%)
**Previous varicose vein operation**	15 (9.6%)	6 (7.4%)
**Treatment**		
**Medication only**	27 (17.3%)	18 (22.2%)
**Compression stocking only**	51 (32.7%)	24 (29.6%)
**Medication + compression stocking**	35 (22.4%)	11 (13.6%)
**Operation**	19 (12.2%)	12 (14.8%)

**Table 2:** Clinical manifestations of patients who presented with unusual varices.

	Gonadal Vein Reflux (+) (n = 156)	Gonadal Vein Reflux (−) (n = 81)
**Telangiectasis or reticular veins**	17 (10.9%)	10 (12.3%)
**Varicose vein** **with leg pain**	80 (51.3%)	40 (49.4%)
**with pelvic pain**	38 (24.4%)	19 (23.5%)
**without symptoms**	5 (3.2%)	2 (2.5%)
**Edema**	16 (10.3%)	9 (11.1%)
**Changes in skin and subcutaneous tissue**	0	1 (1.2%)

**Table 3:** Laterality of limb and gonadal reflux in surgically treated pelvic origin varicose patients.

	Left Gonadal Vein Reflux (n = 18)	Bilateral Gonadal Vein Reflux (n = 1)
Bilateral legs	4	0
Right leg	13	1
Left leg	1	0

## Data Availability

The original contributions presented in the study are included in the article; further inquiries can be directed to the corresponding author.

## References

[1] Kaplan R.M., Criqui M.H., Denenberg J.O., Bergan J., Fronek A. (2003). Quality of life in patients with chronic venous disease: San Diego population study. J. Vasc. Surg..

[2] Smith J.J., Guest M.G., Greenhalgh R.M., Davies A.H. (2000). Measuring the quality of life in patients with venous ulcers. J. Vasc. Surg..

[3] Smith J.J., Garratt A.M., Guest M., Greenhalgh R.M., Davies A.H. (1999). Evaluating and improving health-related quality of life in patients with varicose veins. J. Vasc. Surg..

[4] Korn P., Patel S.T., Heller J.A., Deitch J.S., Krishnasastry K.V., Bush H.L., Kent K.C. (2002). Why insurers should reimburse for compression stockings in patients with chronic venous stasis. J. Vasc. Surg..

[5] Balian E., Lasry J., Coppe G., Borie H., Leroux A., Bryon D. (2008). Pelviperineal venous insufficiency and varicose veins of the lower limbs. Phlebolymphology.

[6] Sutaria R., Subramanian A., Burns B., Hafez H. (2007). Prevalence and management of gonadal venous insufficiency in the presence of leg venous insufficiency. Phlebology.

[7] Hobbs J.T. (2005). Varicose veins arising from the pelvis due to gonadal vein incompetence. Int. J. Clin. Pract..

[8] Taylor H.C. (1949). Vascular congestion and hyperemia: Their effect on structure and function in the female reproductive system. Am. J. Obstet. Gynecol..

[9] Lazarashvili Z., Antignani P.L., Monedero J.L. (2016). Pelvic congestion syndrome: Prevalence and quality of life. Phlebolymphology.

[10] De Maeseneer M.G., Kakkos S.K., Aherne T., Baekgaard N., Black S., Blomgren L., Giannoukas A., Gohel M., de Graaf R., Hamel-Desnos C. (2022). Editor’s choice–European Society for Vascular Surgery (ESVS) 2022 clinical practice guidelines on the management of chronic venous disease of the lower limbs. Eur. J. Vasc. Endovasc. Surg..

[11] Rezaei-Kalantari K., Fahrni G., Rotzinger D.C., Qanadli S.D. (2023). Insights into pelvic venous disorders. Front. Cardiovasc. Med..

[12] McMullin G.M., Smith P.C. (1992). An evaluation of Doppler ultrasound and photoplethysmography in the investigation of venous insufficiency. Aust. N. Z. J. Surg..

[13] Blebea J., Kihara T.K., Neumyer M.M., Blebea J.S., Anderson K.M., Atnip R.G. (1999). A national survey of practice patterns in the noninvasive diagnosis of deep venous thrombosis. J. Vasc. Surg..

[14] Markel A., Meissner M.H., Manzo R.A., Bergelin R.O., Strandness D.E. (1994). A comparison of the cuff deflation method with Valsalva’s maneuver and limb compression in detecting venous valvular reflux. Arch. Surg..

[15] Nicolaides A.N. (1997). Cardiovascular Disease Educational and Research Trust; European Society of Vascular Surgery.

[16] Labropoulos N., Mansour M.A., Kang S.S., Gloviczki P., Baker W.H. (1999). New insights into perforator vein incompetence. Eur. J. Vasc. Endovasc. Surg..

[17] Abai B., Labropoulos N. (2008). Duplex ultrasound scanning for chronic venous obstruction and valvular incompetence. Handbook of Venous Disorders: Guidelines of the American Venous Forum.

[18] O’donnell T.F., Passman M.A., Marston W.A., Ennis W.J., Dalsing M., Kistner R.L., Lurie F., Henke P.K., Gloviczki M.L., Eklöf B.G. (2014). Management of venous leg ulcers: Clinical practice guidelines of the Society for Vascular Surgery^®^ and the American Venous Forum. J. Vasc. Surg..

[19] Eberhardt R.T., Raffetto J.D. (2014). Chronic Venous Insufficiency. Circulation.

[20] Funatsu A., Anzai H., Komiyama K., Oi K., Araki H., Tanabe Y., Nakao M., Utsunomiya M., Mizuno A., Higashitani M. (2019). Stent implantation for May–Thurner syndrome with acute deep venous thrombosis: Acute and long-term results from the ATOMIC (AcTive stenting for May–Thurner Iliac Compression syndrome) registry. Cardiovasc. Interv. Ther..

[21] Suwanabol P.A., Tefera G., Schwarze M.L. (2010). Syndromes associated with the deep veins: Phlegmasia cerulea dolens, May-Thurner syndrome, and nutcracker syndrome. Perspect. Vasc. Surg. Endovasc. Ther..

[22] O’Brien M.T., Gillespie D.L. (2015). Diagnosis and treatment of the pelvic congestion syndrome. J. Vasc. Surg. Venous Lymphat. Disord..

[23] Zamboni P., Mendoza E., Gianesini S. (2018). General Considerations to the Treatment of Pelvic Leak Points, Ch. 8. Saphenous Vein-Sparing Strategies in Chronic Venous Disease.

[24] Francheschi C., Bahnini A. (2005). Treatment of lower extremity venous insufficiency due to pelvic leak points in women. Ann. Vasc. Surg..

[25] Franceschi C. (2008). Anatomie fonctionnelle et diagnostic des points de fuite bulboclitoridiens chez la femme (point C). J. Mal. Vasc..

[26] Gianesini S., Antignani P.L., Tessari L. (2016). Pelvic congestion syndrome: Does one name fit all?. Phlebolymphology.

[27] Whiteley A.M., Taylor D.C., Dos Santos S.J., Whiteley M.S. (2014). Pelvic venous reflux is a major contributory cause of recurrent varicose veins in more than a quarter of women. J. Vasc. Surg. Venous Lymphat. Disord..

[28] Ricci S., Oswald M.A. (2018). Proposal for a protocol for sex hormones level sampling in patients with varices to evidence pelvic reflux. JTAVR.

